# Paramedic Rapid Sequence Intubation (RSI) in a South African Emergency Medical Service (EMS) is effective, but is it safe?

**DOI:** 10.1186/1757-7241-21-S1-S29

**Published:** 2013-05-28

**Authors:** M Gunning, Z Perkins, J Crilly, R von Rahden

**Affiliations:** 1 Inkosi Albert Luthuli Central Hospital, Durban, South Africa; 2 Research Fellow, Trauma Sciences, Queen Mary, University of London, UK; 3 Emergency Department Clinical Network, Queensland, Australia & Griffith University, Australia; 4 Pietermaritzburg Department of Anaesthesia, Critical Care and Pain Management, Grey's Hospital, South Africa

## Background

Early access to critical care interventions may improve outcomes for severely ill and injured patients. A Cochrane review [1] in 2009 reported that evidence supporting prehospital emergency intubation is lacking, but this does not equate to lack of benefit and prehospital RSI remains controversial. South Africa faces unique challenges due to prolonged prehospital times and limited access to physicians. In response to these challenges, the Health Professions Council of South Africa introduced paramedic RSI in 2008. This has been shown to be superior to intubation without paralysis, but a recent meta-analysis concluded that in the absence of prehospital physicians, alternatives to RSI should be strongly considered due to patient safety concerns[2]. This study aimed to identify if RSI performed by paramedics, in the South African prehospital care context is effective and safe.

## Methods

We performed a retrospective observational study of paramedic RSI performed by a national ambulance service, between 12/12/2009 and 12/12/2011. RSI was defined as the administration of Suxamethonium, with or without an induction agent. RSI was performed according to a standard operating procedure following telephonic physician approval. Effectiveness was defined as self-reported successful tracheal intubation. Safety was defined as the absence of an adverse event (AE) (hypoxia or hypotension at handover or reported complications) as a result of the RSI procedure.

## Results

Eighty-six RSI’s were performed during the study period. No failed intubations were reported. Nineteen patients (22%) had an adverse event (AE). Complications included: haemodynamic instability (11.6%), tension pneumothorax (3.5%), difficult intubation (2.3%), low ETCO2 (2.3%), high ETCO2 (1.2%), and bronchospasm (1.2%). 4 of these patients were hypotensive (4.7%) and 2 hypoxic (2.3%) at handover to hospital or helicopter staff. Female gender, helicopter transport and age (<18 years) were significantly associated with an AE on univariate analysis. Female gender (Odds ratio 18.3 (3.46-99.38); p = 0.001) and helicopter transport (Odds ratio 7.24 (1.44-36.32); p = 0.016) remained independently associated with AEs after adjustment for confounders.

## Conclusions

In South African Pre-hospital Care, RSI performed by specially trained paramedics is effective in terms of self-reported success. However, the 1 in 5 AE rate highlights safety concerns. The importance of a robust clinical governance programme to identify problems, refine practice and improve the quality of care is underscored.
